# Intracranial Hemorrhage Detection and Subtype Classification on CT Imaging Using a Large Language Model

**DOI:** 10.7759/cureus.98029

**Published:** 2025-11-28

**Authors:** Nitin Chetla, Shivam Patel, Rahul Kumar, Trisha Naidu, Andrew Bouras, Alireza Tavakkoli, Ethan Waisberg

**Affiliations:** 1 School of Medicine, University of Virginia School of Medicine, Charlottesville, USA; 2 Department of Data Science, University of Virginia School of Medicine, Charlottesville, USA; 3 School of Medicine, University of Massachusetts Chan School of Medicine, Worcester, USA; 4 Department of Computational and Systems Neuroscience, Virginia Polytechnic Institute and State University, Blacksburg, USA; 5 Department of Osteopathic Medicine, Nova Southeastern University Dr. Kiran C. Patel College of Osteopathic Medicine, Clearwater, USA; 6 Department of Computer Science and Engineering, University of Nevada, Reno, USA; 7 Department of Clinical Neurosciences, University of Cambridge, Cambridge, GBR

**Keywords:** artificial intelligence, binary classification, computed tomography, intracranial hemorrhage, large language model, multi-class classification, physionet dataset, subtype classification

## Abstract

Introduction: Accurate detection of intracranial hemorrhage (ICH) and its subtypes on CT scans is critical for timely treatment. While convolutional neural networks have achieved high diagnostic accuracy in this task, the ability of large language models (LLMs) to perform direct medical image interpretation remains largely untested.

Methods: We evaluated a general-purpose multimodal LLM for binary ICH detection and multi-class subtype classification using the publicly available PhysioNet ICH dataset (version 1.3.1, Massachusetts Institute of Technology (MIT), Cambridge, MA). Preprocessed axial slices were grouped into composite images and encoded in base64 for model input. Binary classification distinguished hemorrhage presence versus absence in 75 scans (36 positive, 39 negative). Subtype classification among positive cases included intraventricular, intraparenchymal, subarachnoid, epidural, and subdural hemorrhages. Performance metrics included accuracy, precision, recall, F1 score, exact match accuracy, and Hamming score.

Results: For binary detection, the model achieved an overall accuracy of 0.52, with low recall for hemorrhage-positive cases (0.14) and higher recall for hemorrhage-negative cases (0.87). Subtype performance varied: intraparenchymal hemorrhage reached the highest F1 score (0.57), while epidural hemorrhage showed perfect precision but poor recall (0.14). Exact match accuracy was 0.06, and the Hamming score was 0.54, reflecting partial but inconsistent predictive ability.

Conclusions: The LLM demonstrated limited sensitivity for hemorrhage detection and inconsistent subtype classification, underscoring current constraints of zero-shot application to medical imaging. These findings highlight the need for domain-specific fine-tuning, larger and more diverse datasets, and integration with traditional computer vision methods before clinical deployment.

## Introduction

Accurate identification of intracranial hemorrhage (ICH) and its subtypes on CT imaging is crucial for diagnosis and timely treatment. Misclassification or delayed recognition of hemorrhage can lead to severe morbidity and mortality, particularly in acute emergency settings [1].

Artificial intelligence (AI) methods, particularly convolutional neural networks (CNNs), have demonstrated strong diagnostic performance in this domain. A recent meta-analysis of 58 studies reported a pooled sensitivity of 0.92 and a specificity of 0.94 for deep learning models detecting ICH [2]. Individual systems have achieved near-perfect area under the receiver operating characteristic curve (AUC) values for hemorrhage subtype classification [3], while clinical decision-support platforms such as Aidoc are now FDA-cleared and integrated into practice [4].

Despite these advances, most existing systems are task-specific and depend on large volumes of labeled radiological data. In contrast, large language models (LLMs) adapted for multimodal tasks can interpret both image and text inputs, offering flexible integration of visual reasoning and clinical context [5]. However, the ability of general-purpose LLMs to perform zero-shot interpretation of medical images, without domain-specific fine-tuning, remains largely untested. Evaluating such zero-shot performance is critical to understanding the feasibility, limitations, and potential role of LLMs in future radiology workflows [6,7].

Objective

This study conducts a proof-of-concept evaluation of a general-purpose multimodal LLM for binary ICH detection and subtype classification using the publicly available PhysioNet ICH dataset. Specifically, we aim to benchmark zero-shot LLM performance against established CNN results reported in prior literature. The objective is to assess both the feasibility and current limitations of applying general-purpose LLMs to clinical imaging tasks, providing an evidence-based baseline for future domain-specific fine-tuning and hybrid model development.

## Materials and methods

Dataset and preprocessing

We used the PhysioNet Intracranial Hemorrhage CT Dataset (version 1.3.1, Massachusetts Institute of Technology (MIT), Cambridge, MA) [8], which contains labeled non-contrast head CT scans annotated for hemorrhage presence and five subtypes: intraventricular, intraparenchymal, subarachnoid, epidural, and subdural.

A preprocessing pipeline was implemented in Python (Python Software Foundation, Wilmington, DE). Each volumetric NIfTI file was loaded using NiBabel, and 2D axial slices were extracted. Slices were rotated by 90° for consistent orientation and intensity-windowed to an 8-bit grayscale scale (0-255). Images were saved in PNG format. To address input token limitations, one to four consecutive slices were grouped into composite images in a fixed grid layout and encoded in base64. No compression or resizing was applied.

Classification tasks

Two tasks were evaluated. Binary classification: hemorrhage present vs absent (75 scans; 36 positive, 39 negative); Multi-class subtype classification: among positive scans, classification into intraventricular (n=5), intraparenchymal (n=16), subarachnoid (n=7), epidural (n=21), and subdural (n=4). Several scans contained multiple subtypes.

Model and inference

A general-purpose multimodal LLM was queried through an API interface. Composite CT images were provided as base64-encoded inputs with standardized prompts requesting binary or subtype predictions. The model returned case-level categorical outputs only; no pixel-level heatmaps, overlays, or graded confidence scores were available.

Evaluation metrics

Predictions were compared against dataset labels. Metrics included accuracy, precision, recall, F1 score (harmonic mean of precision and recall), confusion matrices, and additional reliability measures such as balanced accuracy, Cohen’s kappa, and the Matthews correlation coefficient (MCC). These complementary metrics provide further insight into diagnostic reliability, with balanced accuracy accounting for class distribution, MCC summarizing overall correlation between predictions and ground truth, and kappa quantifying agreement beyond chance. For multi-label subtype classification, we calculated exact match accuracy (proportion of cases where the predicted subtype set exactly matched the ground truth) and the Hamming score (fraction of correctly predicted labels over all labels). Confidence intervals (95%) were estimated using Wilson score intervals for binary classification metrics and nonparametric bootstrapping for F1 scores.

## Results

Binary classification

**Figure 1:**
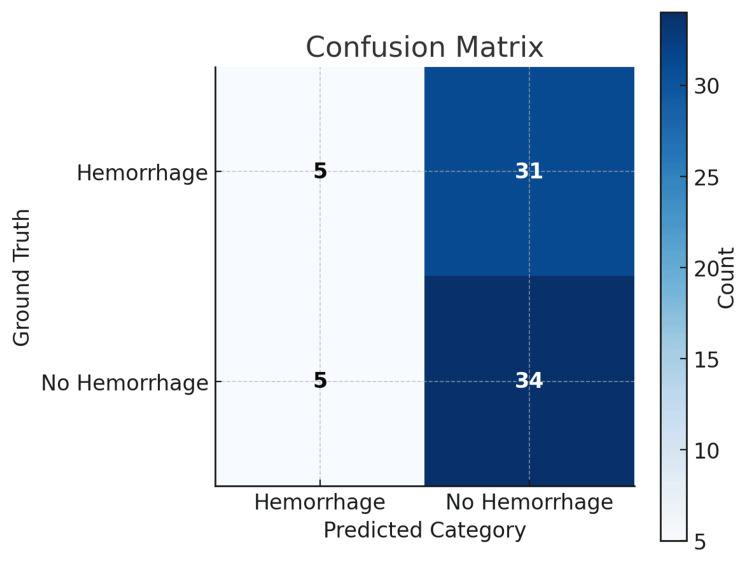
Confusion matrix for binary intracranial hemorrhage detection The matrix compares predicted versus ground truth classifications for hemorrhage presence (positive) and absence (negative). Cell values represent the number of cases, with darker shading indicating higher counts.

For binary detection of hemorrhage, the model achieved an overall accuracy of 0.52. Performance metrics are summarized in Table 1. For hemorrhage-positive cases, precision was 0.50, recall was 0.14, and the F1 score was 0.22, reflecting limited sensitivity. For hemorrhage-negative cases, precision was 0.52, recall was 0.87, and the F1 score was 0.65. The confusion matrix (Figure 1) illustrates the strong bias toward negative predictions, with the majority of positive cases misclassified as absent.

**Table 1:** Performance metrics for binary classification of hemorrhage presence Performance metrics for binary classification of hemorrhage presence (n = 75 scans; 36 positive, 39 negative). Metrics include precision, recall, and F1 score for each class. Macro average (unweighted mean across classes) and weighted average (mean across classes weighted by support) summarize performance across classes. Overall accuracy for the task was 0.52. Additional reliability metrics included balanced accuracy (0.50), Matthews correlation coefficient (MCC, -0.01), and Cohen’s kappa (0.03).

Metric	Precision	Recall	F1 score	Support
Hemorrhage Present (A)	0.5	0.14	0.22	36
No Hemorrhage Present (B)	0.52	0.87	0.65	39
Macro Average	0.51	0.51	0.44	75
Weighted Average	0.51	0.51	0.44	75

Subtype classification

Among hemorrhage-positive cases, performance varied across subtypes (Table 2). Intraparenchymal hemorrhage achieved the highest F1 score (0.57), supported by relatively balanced precision (0.46) and recall (0.75). Intraventricular hemorrhage showed high recall (0.80) but poor precision (0.15), resulting in an F1 score of 0.25. Epidural hemorrhage demonstrated perfect precision (1.00) but low recall (0.14). Subarachnoid hemorrhage achieved moderate recall (0.71) but limited precision (0.28). Subdural hemorrhage showed low precision (0.17), recall (0.25), and F1 score (0.20).

**Table 2:** Performance metrics for multi-class subtype classification among hemorrhage-positive cases Performance metrics for multi-class subtype classification among hemorrhage-positive cases (n = 36 scans). Subtypes included intraventricular (n = 5), intraparenchymal (n = 16), subarachnoid (n = 7), epidural (n = 21), and subdural (n = 4). Metrics include precision, recall, and F1 score for each subtype, with macro and weighted averages. Overall exact match accuracy was 0.06, while the Hamming score was 0.54, indicating partial but inconsistent predictive ability.

Metric	Precision	Recall	F1-score	Support
Intraventricular	0.15	0.8	0.25	5
Intraparenchymal	0.46	0.75	0.57	16
Subarachnoid	0.28	0.71	0.4	7
Epidural	1	0.14	0.25	21
Subdural	0.17	0.25	0.2	4
Macro Average	0.41	0.53	0.33	53
Weighted Average	0.6	0.47	0.36	53

**Figure 2:**
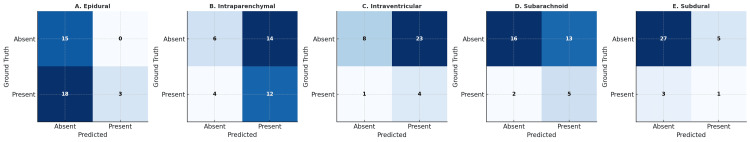
Confusion matrices for hemorrhage subtype classification (A) Epidural; (B) Intraparenchymal; (C) Intraventricular; (D) Subarachnoid; and (E) Subdural hemorrhage. Each matrix compares predicted versus ground truth classifications, with counts of correctly and incorrectly classified cases displayed in each cell. Darker shading indicates higher counts.

Aggregate performance across subtypes was modest, with an exact match accuracy of 0.06, indicating that the model rarely predicted the complete correct set of subtypes for a scan. The Hamming score was 0.54, suggesting partial but inconsistent predictive ability. Figure 2 provides confusion matrices for each subtype, highlighting frequent misclassification between absent and present categories.

## Discussion

This proof-of-concept study demonstrates that a general-purpose multimodal LLM achieved limited sensitivity for ICH detection and inconsistent subtype classification, with modest overall accuracy and very low exact match rates. The model frequently defaulted toward negative predictions, producing false negatives that undermine its potential utility in acute care.

By comparison, CNN-based systems trained specifically on ICH detection report much stronger performance. Meta-analyses and benchmark studies consistently show AUCs above 0.95, with high sensitivity and specificity for both detection and subtyping [1-3]. Clinical AI tools such as Aidoc demonstrate reliable detection in real-world emergency workflows [4]. The disparity between these domain-tuned systems and zero-shot LLM performance underscores the importance of specialized training.

**Figure 3:**
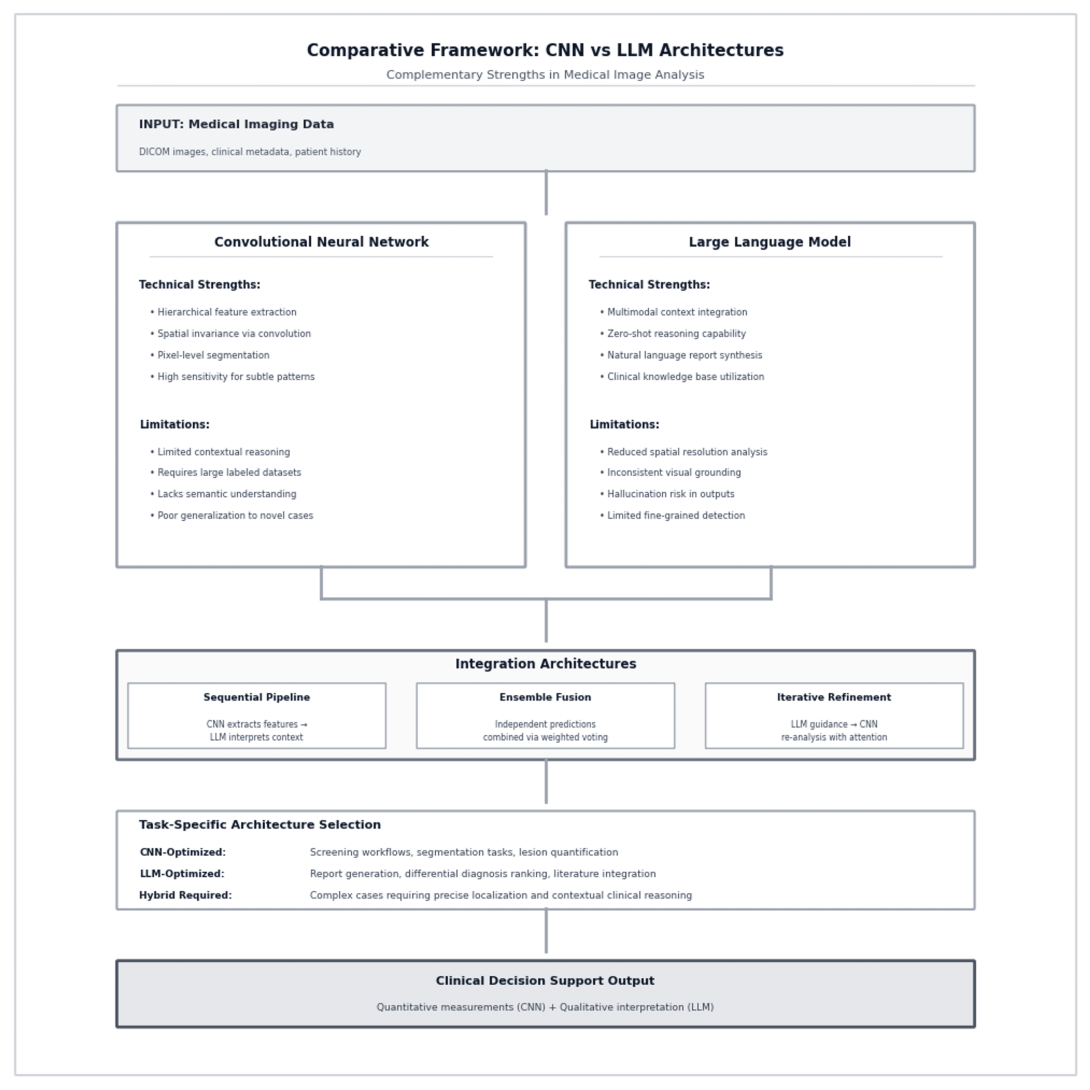
Comparative framework for CNN and LLM architectures in intracranial hemorrhage analysis The diagram illustrates complementary strengths and limitations of convolutional neural networks (CNNs) and large language models (LLMs) when applied to medical image analysis. CNNs provide hierarchical feature extraction, spatial invariance, and pixel-level segmentation with high sensitivity to subtle patterns but are limited in contextual reasoning and generalization. LLMs enable multimodal integration, zero-shot reasoning, and natural language report synthesis, yet lack spatial resolution and visual grounding. Integration architectures include sequential pipelines (CNN feature extraction followed by LLM interpretation), ensemble fusion (independent predictions combined via weighted voting), and iterative refinement (LLM-guided CNN re-analysis). Task-specific architecture selection highlights CNNs as optimal for screening and segmentation workflows, LLMs for report generation and contextual reasoning, and hybrid systems for complex cases requiring both quantitative precision and semantic interpretation.

The divergent performance characteristics observed in this study reflect fundamental architectural differences between CNNs and LLMs. CNNs excel at hierarchical feature extraction through convolutional layers, enabling precise spatial localization and sensitivity to subtle pixel-level patterns, capabilities that translate directly to high diagnostic accuracy in ICH detection [2,3]. However, their task-specific training and limited semantic reasoning restrict flexibility in clinical workflows. Conversely, LLMs demonstrate strength in multimodal context integration and zero-shot reasoning but lack the spatial resolution and visual grounding necessary for reliable hemorrhage detection, as evidenced by our results. These complementary profiles suggest potential value in hybrid architectures that strategically leverage each model’s strengths while compensating for the other’s limitations. Figure 3 illustrates potential integration strategies: sequential pipelines where CNN feature extraction informs LLM interpretation, ensemble fusion combining independent predictions, and iterative refinement using LLM guidance to direct CNN attention. Task-specific optimization would determine architecture selection, with CNNs preferred for screening and segmentation workflows, LLMs for report generation and differential ranking, and hybrid approaches for complex cases requiring both precise localization and contextual clinical reasoning.

LLMs nonetheless offer flexibility in multimodal reasoning, which could be leveraged in hybrid frameworks that integrate text, clinical metadata, and imaging [5,6]. In such a pipeline, CNNs (or vision transformers) can provide pixel-level feature extraction, segmentation maps, or probability scores for hemorrhage and subtypes, while LLMs can contextualize these structured outputs by combining them with clinical history, radiology reports, and differential diagnoses. This complementary framework would allow CNNs to overcome the LLM’s limited sensitivity by supplying accurate visual signals, while LLMs mitigate CNN task-specific rigidity by enabling interpretability-rich reasoning and workflow integration [9].

Furthermore, concerns remain about hallucinations, dataset bias, and legal liability when applied in high-stakes diagnostic contexts [10-12]. These risks highlight the need for transparency, safeguards against perpetuating healthcare disparities, and clear delineation of medico-legal accountability before such systems can be deployed in acute care environments.

Our dataset was relatively small and deliberately balanced, which facilitates interpretability but diverges from real-world prevalence. Subtype diversity was limited, and labels derived from metadata may not fully capture nuanced radiologist consensus. The absence of pixel-level localization, confidence scores, and prospective validation restricts clinical applicability. In addition, the use of a single public dataset limits generalizability, and zero-shot inference without domain-specific fine-tuning may underestimate achievable performance. These limitations underscore that our results should be interpreted cautiously and viewed as a proof-of-concept benchmark rather than evidence of clinical readiness.

Future directions include (1) fine-tuning multimodal LLMs on large annotated radiology datasets, (2) expanding subtype coverage with more balanced class distributions, (3) developing hybrid systems that integrate CNNs for image parsing with LLMs for reasoning and reporting, and (4) validating prospective performance across institutions. Emerging explainability frameworks, such as segmentation-aware or reasoning-augmented architectures, may further bridge the gap between general-purpose AI and clinical needs [13-15].

## Conclusions

A general-purpose multimodal LLM demonstrated modest performance in binary ICH detection and highly variable accuracy in subtype classification. Its low sensitivity for hemorrhage-positive cases highlights the risks of applying zero-shot LLMs directly to medical imaging tasks. Significant improvements, through domain-specific fine-tuning, integration with traditional vision models, and prospective validation, are required before clinical application. At present, LLMs in radiology should be considered exploratory adjuncts rather than standalone diagnostic tools.

## References

[1] Karamian A, Seifi A (2025). Diagnostic accuracy of deep learning for intracranial hemorrhage detection in non-contrast brain CT scans: a systematic review and meta-analysis. J Clin Med.

[2] Wang X, Shen T, Yang S (2021). A deep learning algorithm for automatic detection and classification of acute intracranial hemorrhages in head CT scans. Neuroimage Clin.

[3] Lee JY, Kim JS, Kim TY, Kim YS (2020). Detection and classification of intracranial haemorrhage on CT images using a novel deep-learning algorithm. Sci Rep.

[4] Zia A, Fletcher C, Bigwood S (2022). Retrospective analysis and prospective validation of an AI-based software for intracranial haemorrhage detection at a high-volume trauma centre. Sci Rep.

[5] Jiang F, Jiang Y, Zhi H (2017). Artificial intelligence in healthcare: past, present and future. Stroke Vasc Neurol.

[6] Wang Y, Zeng Y, Chen K, Meng C, Pan C, Tang Z (2025). Zero-shot multimodal large language models vs. supervised deep learning: a comparative study on CT-based intracranial hemorrhage subtyping. arXiv.

[7] Soenksen LR, Ma Y, Zeng C (2022). Integrated multimodal artificial intelligence framework for healthcare applications. NPJ Digit Med.

[8] Hssayeni MD, Croock MS, Salman AD, Al-khafaji HF, Yahya ZA, Ghoraani B (2020). Intracranial hemorrhage segmentation using a deep convolutional model. Data.

[9] Zhang H, Yang YF, Song XL, Hu HJ, Yang YY, Zhu X, Yang C (2024). An interpretable artificial intelligence model based on CT for prognosis of intracerebral hemorrhage: a multicenter study. BMC Med Imaging.

[10] Celi LA, Cellini J, Charpignon ML (2022). Sources of bias in artificial intelligence that perpetuate healthcare disparities-a global review. PLOS Digit Health.

[11] Recht M, Bryan RN (2017). Artificial intelligence: threat or boon to radiologists?. J Am Coll Radiol.

[12] Rosic A (2024). Legal implications of artificial intelligence in health care. Clin Dermatol.

[13] Topol EJ (2019). High-performance medicine: the convergence of human and artificial intelligence. Nat Med.

[14] Houssein EH, Gamal AM, Younis EMG, Mohamed E (2025). Explainable artificial intelligence for medical imaging systems using deep learning: a comprehensive review. Cluster Comput.

[15] Liu X, Faes L, Kale AU (2019). A comparison of deep learning performance against health-care professionals in detecting diseases from medical imaging: a systematic review and meta-analysis. Lancet Digit Health.

